# Effect of washing on the post-thaw quality of cryopreserved ram epididymal spermatozoa

**DOI:** 10.14202/vetworld.2016.519-523

**Published:** 2016-05-27

**Authors:** Touqeer Ahmed, Rafiqul Islam, Farooz Ahmad Lone, Asloob Ahmad Malik

**Affiliations:** Division of Animal Reproduction, Gynaecology and Obstetrics, Faculty of Veterinary Sciences and Animal Husbandry, Sher-e-Kashmir University of Agricultural Sciences & Technology of Kashmir, Shuhama, Alusteng, Srinagar, Jammu & Kashmir, India

**Keywords:** cauda epididymis, centrifugation, processing, ram, spermatozoa, washing

## Abstract

**Aim:**

The aim of the study was to evaluate the effect of washing on the post-thaw quality of ram cauda epididymal spermatozoa (P1: Unwashed, P2: Washed).

**Materials and Methods:**

Fresh testicles of adult healthy slaughtered rams were collected and transported to the laboratory in an ice chest, where they were weighed, and cauda epididymides were separated. These cauda epididymides were used for recovery of spermatozoa in tris-citric acid fructose buffer by incision method. Spermatozoa samples showing ≥70% progressive motility were pooled and processed further. The mean values (±standard error) of various parameters such as the percentage of sperm motility, live sperm, intact acrosome, and hypo-osmotic swelling test (HOST) reacted spermatozoa were recorded.

**Results:**

In this experiment, the percent sperm motility, live spermatozoa, and intact acrosome both at pre-freeze and post-thaw were higher (p>0.05) in P1 than P2. However, the post-thaw percent HOST reacted spermatozoa was slightly higher (p>0.05) for P2 than P1.

**Conclusion:**

Washing of cauda epididymal spermatozoa has no significant adverse effect on the quality during cryopreservation. Therefore, this processing method can be applied wherever necessary before the extension of the recovered spermatozoa sample in different ram extenders.

## Introduction

The use of cryopreserved sperm in assisted reproductive techniques has become an indispensable tool for breeding management in domestic animals [1]. So, being an important technique semen cryopreservation is used for the application of assisted reproduction such as artificial insemination and *in vitro* fertilization, which contribute to increased production and genetic selection [2]. However, during cryopreservation massive ultrastructural, biochemical, and functional damages occur on the spermatozoa due to the stress and temperature changes, which result in decreased motility and viability of the spermatozoa leading to decreased fertility, which are the main drawbacks of this technique [3,4].

Several researchers have studied the effect of different washing solutions and centrifugations regimes in different species to find out a proper method for improving the quality of cryopreserved semen. In buck semen cryopreservation, the centrifugation regimes and washing solutions used were 600 ×*g* for 10 min with Krebs–Ringer phosphate plus sodium citrate [5], 1200 ×*g* for 15 min with tris-citric acid glucose (TCG) buffer [6], 1500 ×*g* for 10 min with TCG [7], and 1000 ×*g* for 10 min with Ringer’s lactate [8]. Earlier studies on the influence of different centrifugation regimes (400, 800, 1600, and 2400 ×*g*) on boar spermatozoa have reported that the use of short-term centrifugation with a relative high g-force (2400 ×*g* for 3 min) caused a positive effect on its cryosurvival [9]. Webb and Dean [10] described that post-thaw motility of frozen stallion sperm was not different between centrifugation treatments (700 ×*g* for 15 min, 600 ×*g* for 12 min, and 400 ×*g* for 7 min). Sarıozkan *et al*. [8] described that a high fertility rate, with or without centrifugation/washing, buck semen could be achieved with the Bioxcell extender.

Therefore, the results presented in the literature about washing of spermatozoa are quite variable [11] and studies evaluating the effects of washing or centrifugation on the cryopreservation of cauda epididymal spermatozoa are still very limited. Hence, to overcome this variability, the researchers should work to reach a consensus that generally addresses accepted practices for the effects of centrifugation in cauda epididymal sperm cryopreservation. Therefore, this study was carried out to study the effect of washing on the quality of cauda epididymal spermatozoa before freezing and after thawing.

## Materials and Methods

### Ethical approval

No ethical approval was necessary to pursue this work, because no live animal was involved while carrying out the whole study. Samples were collected from government approved slaughter house.

### Extender used

Tris-citric acid fructose (TCF) egg yolk extender was used for the extension of the spermatozoa and the effect of washing of spermatozoa on post-thaw quality was evaluated.

### Collection of testicles

About 18 intact testicles of freshly slaughtered adult healthy rams were collected from government approved abattoir in Rainawari Srinagar city. They were then transported to the semen processing laboratory at Sperm station, Frozen semen project, Ranbirbagh, Ganderbal, in an ice chest, where further processing of these testicles and recovery of spermatozoa was done.

### Processing of testicles

In the laboratory, the testicles were cleaned by removing the additional tissues. Then, biometric measurements of the testicles were done, including testicular, whole epididymal and cauda epididymal weight, on an electronic balance. All the values obtained from the above procedure were recorded simultaneously.

### Collection of spermatozoa

The spermatozoa were recovered from the cauda epididymis in tris buffer (Table-1) using incision method [12,13].

**Table-1:** Composition of TCF buffer.

Ingredients	Quantity
Tris (hydroxyl methylamino methane)	3.028 g
Citric acid monohydrate	1.70 g
Fructose	1.25 g
Distilled Water ad	100 ml

TCF=Tris-citric acid fructose

### Initial evaluation of spermatozoa

After recovery of the spermatozoa from the cauda epididymis in petri-dish, the concentration and progressive motility of each sample were determined. The concentration was determined using a photometer (IMV-France). Then, the progressive motility was determined [14], and samples showing motility ≥70% were pooled and subsequently divided into two aliquots.

### Washing of spermatozoa

Two aliquots were made from the pooled cauda epididymal spermatozoa. Spermatozoa of one aliquot (P2) were washed (centrifuged) twice using tris buffer, and the other aliquot (P1) was extended without washing (control). The centrifugation of P2 was carried out at 855 g for 10 min in centrifuge machine (REMI Laboratory R4C, Maharashtra, India). After centrifugation, the supernatant was discarded, and the remaining spermatozoa were diluted with TCF extender.

### Extension of the sample

Each aliquot was extended in TCF egg yolk extender (Table-2) [15].

**Table-2:** Composition of TCF extender.

Ingredients	Quantity
TCF buffer	72 ml
Glycerol	8 ml
Egg yolk	20 ml
Penicillin G sodium	80000 i.u
Streptomycin sulfate	100 mg

TCF=Tris-citric acid fructose

### Quality parameters of extended spermatozoa

The quality of the extended cauda epididymal sperm samples was evaluated for percent progressive motility. Then, live sperm percentage was determined using Eosin–Nigrosin differential staining technique as per standard procedures [16], the percentage of intact acrosome was estimated using standard Giemsa staining technique [17] and hypo-osmotic swelling test (HOST) was carried out as per Vasquez *et al*. [18].

### Preservation of the sample

Six cauda epididymal sperm samples from both groups that showed ≥70% motility were extended. The extended semen was filled and sealed in French mini straws (0.25 ml) and subsequently printed, in automatic filling sealing and printing machine (IMV-France). The straws were then equilibrated at 4°C for 4 h. The equilibrated straws were subjected to rapid vapor freezing in Biological Programmable Freezer, and finally, the frozen straws were placed into liquid nitrogen and stored in cryocans until further post-thaw analysis.

### Post-thaw analysis

The frozen semen straws were thawed in warm water at 37°C for 15 s, and the post-thaw quality of each sample was determined by the parameters and procedures already discussed.

### Statistical analysis

The data obtained in the study were analyzed statistically using t-test. The data pertaining to sperm quality between pre-freeze and post-thaw were compared by paired samples t-test with the help of statistical software SPSS version 16. The level of significance was set at p<0.05. The data are presented in the tables as mean±standrad error of mean.

## Results

The mean weight (g) of the testicles utilized for recovery of spermatozoa in this study was 128.67±4.69 (116.60-140.73). The average weight (g) of the whole epididymis was 17.48±1.14 (14.54-20.43) and that of cauda epididymis was 8.35±0.21 (7.82-8.89). The mean spermatozoa concentration (million/ml) of the pooled sample was 1333±142.71 (966.16-1699.84). The recovery of the spermatozoa from the cauda epididymides was done in TCF buffer. Each of the 6 pooled samples was obtained from the spermatozoa recovered from three cauda epididymides. Each pool was divided into two aliquots, out of which, one aliquot was extended without washing (P1), and another was washed using TCF buffer (P2). Both the aliquots were extended in TCF extender and cryopreserved.

### Sperm motility

The percent sperm motility at pre-freeze was higher (p>0.05) for P1 (77.50±1.12) than P2 (75.00±1.82). At post-thaw, the percent sperm motility was also higher (p>0.05) for P1 (53.33±3.07) than P2 (40.00±6.45). The percentage of sperm motility for both the processing methods (P1 and P2) decreased significantly (p>0.05) from pre-freeze to post-thaw (Table-3).

**Table-3:** Quality parameters in washed and unwashed sperm samples at pre-freeze and post-thaw (mean±SEM).

Parameters (%)	Processing methods	Pre-freeze	Post-thaw
Sperm motility	Unwashed (P1)**	77.50±1.12^a^	53.33±3.07^b^
	Washed (P2)**	75.00±1.82^a^	40.00±6.45^b^
	Overall**	76.25±1.09^a^	46.67±3.96^b^
Live sperm	Unwashed (P1)**	83.06±1.23^a^	65.96±4.79^b^
	Washed (P2)**	81.57±1.12^a^	54.98±9.12^b^
	Overall**	82.31±0.83^a^	60.47±5.18^b^
Intact acrosome	Unwashed (P1)**	92.38±0.83^a^	74.63±4.96^b^
	Washed (P2)**	90.59±0.87^a^	70.89±6.45^b^
	Overall**	91.49±0.64^a^	72.76±3.92^b^
HOST reacted spermatozoa	Unwashed (P1)**	86.36±0.87^a^	57.16±2.72^b^
	Washed (P2)**	84.87±0.37^a^	59.95±2.73^b^
	Overall**	85.62±0.50^a^	58.55±1.88^b^

Means with different superscripts in a row (a,b) differ significantly (**p<0.01). HOST=Hypo-osmotic swelling test, SEM=Standard error of mean

### Live sperm percentage

The live sperm percentage was higher (p>0.05) for unwashed (P1) cauda epididymal spermatozoa both at pre-freeze and post-thaw than washed sample (P2). The post-thaw live sperm percentage was 65.96±4.79 and 54.98±9.12 for P1 and P2, respectively. The live sperm percentage decreased significantly (p>0.05) from pre-freeze to post-thaw for both the processing methods (Table-3).

### Percent intact acrosome

The intact acrosome percentage did not differ significantly (p>0.05) between the processing methods (P1 and P2) both at pre-freeze and post-thaw. However, the intact acrosome percentage was higher (p>0.05) in P1 (74.63±4.96) than P2 (70.89±6.45) at post-thaw. The intact acrosome percentage also declined significantly (p>0.05) from pre-freeze to post-thaw (Table-3).

### HOST reacted spermatozoa

The percentage of HOST reacted spermatozoa did not differ significantly (p>0.05) between the processing methods (P1 and P2) both at pre-freeze and post-thaw. However, the percentage of HOST reacted spermatozoa declined significantly (p>0.05) from pre-freeze to post-thaw (Table-3).

## Discussion

In the wild, threatened or endangered species of animals may die unexpectedly, either naturally or due to poaching. Recovery and cryopreservation of the epididymal spermatozoa would be one useful way to rescue the germplasm of these dead and valuable animals, and then use them to preserve endangered species. However, cryopreservation causes ultrastructural, biochemical, and functional damages on spermatozoa due to the processing stress and temperature changes resulting in decreased motility and viability with the subsequent decrease in its fertility potential. Studies on the effects of washing with an isotonic buffer using centrifugation on cauda epididymal spermatozoa during cryopreservation are still very limited. Moreover, the results presented in the literature are quite variable. Hence, to overcome this variability, the researchers should endeavor to reach a consensus on the effects of centrifugation on the cauda epididymal spermatozoa during cryopreservation. Therefore, this experiment was carried out to analyze the effects of washing on the quality of cauda epididymal spermatozoa at post-thaw.

The mean percent sperm motility, viable spermatozoa, and intact acrosome were slightly higher (p>0.05) for the unwashed sample, i.e. sample without centrifugation (P1) both at pre-freeze and post-thaw than the washed spermatozoa (P2). Similarly, Hoogewijs *et al*. [19] also reported no overall effect of centrifugation for the percentage of membrane-intact sperm cells, percentage acrosome-intact sperm cells, total motility, and progressive motility. The percentage of HOST reacted spermatozoa was higher (p>0.05) at pre-freeze; however, at post-thaw, the figure was higher (p>0.05) in the case of washed spermatozoa. The figures for the quality parameters were within the acceptable limit for both the processing methods. The slightly lower percentage of sperm motility, live sperm, and intact acrosome in the P2 at pre-freeze might be due to the stress occurred in the spermatozoa owing to the double centrifugation immediately after recovery and before extension in the final extender and cryopreservation and same has been reflected during the post-thaw. These results are in agreement with the findings of Lone *et al*. [13] in ram epididymal spermatozoa and Islam *et al*. [20] in case of washed goat spermatozoa after holding. Washing of ram spermatozoa is also known to cause damage to the plasma membrane and the acrosome [21].

Decrease in sperm motility of ejaculated goat spermatozoa owing to double centrifugation after holding at 24°C for 1, 3, and 5 h has been reported [20]. However, they reported the beneficial effects of washing of spermatozoa with tris buffer by centrifugation without holding and subsequent preservation in tris extender at 5°C. The beneficial effect of washing on goat ejaculated spermatozoa was obtained due to the removal of the harmful egg yolk coagulating enzyme before their preservation in egg yolk based extender [22,20] which does not exist in ram cauda epididymal spermatozoa sample. Better quality during preservation of unwashed cauda epididymal ram spermatozoa in egg yolk citrate extender has also been reported [23]. Being the values in respect of all the parameters within the acceptable limits and the non-observance of any significant difference between the two processing methods, it could be concluded that epididymal spermatozoa could be cryopreserved with or without centrifugation depending on the need of the protocol or experimental study and washing of cauda epididymal spermatozoa has no significant adverse effect on the quality during cryopreservation. Therefore, this processing method can be applied wherever necessary before the extension of the recovered spermatozoa sample in different extenders.

## Conclusion

From the above study, it can be concluded that centrifugation as method of washing cauda epididymal spermatozoa has no significant adverse effect on the quality during cryopreservation. Therefore, this processing method can be applied wherever necessary before the extension of the recovered spermatozoa sample in different extenders.

## Authors’ Contributions

This study is the part of M.V.Sc thesis work of TA, which was conducted under the guidance of RI. TA conducted the whole study and designed the manuscript under the supervision of RI. FAL and AAM revised the manuscript and helped in statistical analysis. All authors read and approved the final manuscript.
